# rs401502 and rs11575934 Polymorphisms of the IL-12 Receptor Beta 1 Gene are Protective Against Colorectal Carcinogenesis

**DOI:** 10.3389/fgene.2022.864419

**Published:** 2022-05-13

**Authors:** Refka Jelassi, Sabrine Dhouioui, Hamza Ben Salah, Nasreddine Saidi, Nabiha Mzoughi, Radhia Ammi, Aida Bouratbine, Karim Aoun, Ines Zidi, Hanen Chelbi

**Affiliations:** ^1^ Laboratory of Medical Parasitology, Biotechnology, and Biomolecules, Pasteur Institute of Tunis, University Tunis El Manar, Tunis, Tunisia; ^2^ Faculty of Sciences Bizerte, University of Carthage, Tunis, Tunisia; ^3^ Laboratory Microorganisms and Active Biomolecules, Faculty of Sciences of Tunis, University Tunis El Manar, Tunis, Tunisia; ^4^ External Consultants Service Pasteur Institute of Tunis, Tunis, Tunisia

**Keywords:** colorectal cancer, IL-12RB1, rs401502, rs11575934, single nucleotide polymorphism (SNP)

## Abstract

**Background:** Colorectal cancer (CRC) is a major public health problem worldwide and in Tunisia. It ranks among the main cancers in terms of incidence and cancer-related cause of death. Its pathogenesis is currently considered to be multifactorial involving genetic and environmental factors. Recent studies have suggested that the gene encoding the β1 subunit of the IL-12 receptor, an important pro-inflammatory cytokine of the anti-tumor response, could be involved in the susceptibility to inherited CRC. Hence, it would be interesting to study the role of single nucleotide polymorphisms (SNPs) within the IL-12RB1 gene (rs401502 and rs11575934) in CRC susceptibility.

**Aim:** Our purpose was to assess whether genetic variants IL-12RB1 +1196G/C (rs401502) and IL-12RB1 +705A/G (rs11575934) within the IL-12RB1 gene are associated with the sporadic CRC risk.

**Methods:** A total of 110 Tunisian patients with sporadic CRC and 141 healthy control subjects were included in this study. Genotyping was performed by high-resolution melting (HRM) analysis. All results were confirmed by direct DNA sequencing or PCR-RFLP methods. Later, the allele frequencies and genotype distribution were established and compared between the control group and CRC patients.

**Results:** The obtained results showed that the two target SNPs were in Hardy–Weinberg equilibrium (HWE) in both patients and controls. Minor allele frequencies of rs401502 SNP were 16.4% in CRC cases and 23.8% in controls. Mutant allele of rs11575934 SNP was present with 21.4% in CRC patients and 29.8% in control group. An association study showed a significant association of two target polymorphisms with CRC, according to the dominant genetic model with OR = 0.577, 95% CI = [0.343 to 0.972], *p* = 0.038 and OR = 0.547, 95% CI = [0.328 to 0.911], *p* = 0.02, respectively.

**Conclusion:** In this study, we found, for the first time, a potential protective effect of two SNPs in the IL-12RB1 gene, namely rs401502 and rs11575934, in sporadic colorectal cancer in Tunisians.

## Introduction

Colorectal cancer (CRC) is one of the most prevalent gastrointestinal malignancies (Araghi et al., 2019). It is the third most common cancer diagnosed in both men and women and the second leading cause of cancer deaths worldwide in 2020 (Sung et al., 2021). Tunisia is also highly affected by this disease with an increase in the incidence these last years (Khiari et al., 2017). CRC is a complex disease that involves many factors in its tumorigenesis, namely, hereditary, genetic, and environmental factors (Lucas et al., 2017; Behrens et al., 2018; Balhareth et al., 2019; Wong et al., 2019; McNabb et al., 2020). Several studies have been carried out in order to investigate such risk factors and therefore to better understand the mechanisms of its genesis, improve early detection, and thus increase the effectiveness of treatment (Seo and Cairns 2018).

Cytokines play a major role in modulating the immune response and in organizing the control of pathological cells, including cancer cells. These cytokines act on immune cells by modulating their activation, differentiation, and multiplication (Dinarello, 2007). The presence of variations in genes encoding cytokines could impact their expression, structure, regulation, and function, thus promoting the development of pathologies. Therefore, studying the role of genetic variations within genes encoding cytokines is essential to better understand the carcinogenesis process (Stanilova, 2012). Several genetic polymorphisms of genes encoding cytokines and their receptors have been studied for their association with colon carcinogenesis, in particular IL-6, IL1β, IL-1RN, IL-10, IL17, and TNF α (Sun et al., 2015; Bedoui et al., 2018; Ibrahimi et al., 2018; Mirjalili et al., 2018; Shi et al., 2018; Huang et al., 2019; Liu et al., 2020).

Interleukin-12 (IL-12) is a pro-inflammatory cytokine secreted by activated phagocytic and dendritic cells. It plays an important role in promoting Th1-type immune responses and cell-mediated immunity (Trinchieri 1998; Miteva et al., 2009). IL-12 exerts its action by binding to its receptor (IL-12R) with high affinity. This receptor is mainly expressed by T cells and natural killer (NK) cells. It is composed of two distinct subunits IL-12Rβ1 and IL-12Rβ2 and contributes to both IL-12/IL-23 signaling pathways (Presky et al., 1996). The gene encoding IL-12Rβ1 is located on chromosome 19 at position 31.1 (19p31.1), and it has 17 exons (Yamamoto et al., 1997). A total of 70 SNPs have been reported in the IL-12RB1 gene, of which the missense mutation is the most frequent type (van de Vosse et al., 2005). Certain mutations in the IL-12RB1 gene generate an inactive IL-12Rβ1 protein. This deficiency abolishes both IL-12 and IL-23 signaling, which play a crucial role in the cytokine signaling pathways (Núñez-Marrero 2020). In fact, several studies have been carried out to explore the association between IL-12RB1 polymorphisms and certain diseases such as cervical cancer (Hussain et al., 2013), breast cancer (Quan et al., 2014), and tuberculosis (Altare et al., 2001; Remus 2004; de Beaucoudrey et al., 2010; Boisson-Dupuis et al., 2011; Alinejad Dizaj et al., 2018; Zhou et al., 2019). Hence, the IL-12RB1 gene appears interesting as a candidate gene for testing susceptibility to cancers.

Recently, Belhadj and collaborators showed an association between the allele frequencies of IL-12RB1 polymorphisms in patients with hereditary CRC in a family study (Belhadj et al., 2020). These results were based on previous studies that identified IL-12RB1 as a potential candidate gene for its biological function (Chubb et al., 2016a). The rs401502 and rs11575934 SNPs are located in a protein-coding region of the IL-12RB1 gene within exon 10 and exon7, respectively (de Beaucoudrey et al., 2010). These are missense mutations characterized by a substitution of a single nucleotide with another, resulting in an amino acid sequence change (IL-12RB1+1196 G/C; G378R; ID SNP NCBI: rs401502 and IL-12RB1+705 A/G; Q214R; ID SNP NCBI: rs11575934). These mutations lead to the absence of IL-12Rβ1 expression on the surface of activated T and NK cells or, more rarely, they give rise to a non-functional protein (van de Vosse et al., 2013). To the best of our knowledge, there were no studies that involved the association between these two mutations and sporadic colorectal cancer susceptibility. These polymorphisms lead to the absence of the expression of this protein on the surface of activated T and NK cells or, more rarely, they give rise to a non-functional protein (van de Vosse et al., 2013).

In this study, we investigated for the first time the association between two single nucleotide polymorphisms (SNPs) within the IL-12RB1 gene, namely, IL-12RB1 +1196G/C (G378R, NCBI SNP ID: rs401502) and IL-12RB1 +705A/G (Q214R, NCBI SNP ID: rs11575934), and the colorectal cancer susceptibility in a Tunisian cohort.

## Materials and Methods

### Study Subjects

We performed a case-control association study including 110 Tunisian patients with sporadic CRC and 141 healthy volunteers as a control group. Cases were confirmed by colonoscopy and histology, and the tumors were classified according to the tumor–node–metastasis (TNM) classification. Healthy subjects with previous gastrointestinal problems (inflammatory bowel diseases or others) were excluded from this study. Our study populations were recruited from Salah-Azaïz Institute and from external consultants’ service, Pasteur Institute of Tunis.

All subjects included in this study agreed to participate and signed an informed consent form. All collected data were anonymized, and no personal data were used. Ethical approval was obtained from the Ethics Committee of the Salah-Azaïz Institute of Tunis (reference: ISA/2016/03).

### Biological Samples and DNA Extraction

A whole blood sample (5 ml) was collected on an EDTA tube for both study populations. DNA extraction was performed using the salting-out method described by Miller et al. (Miller et al., 1988). The DNA concentration and purity were measured by using the NanoDrop 2000 spectrophotometer (Thermo Fisher). All DNA working concentrations were adjusted to 10 ng/μL.

### SNP Genotyping

Two SNPs of interest (rs401502 and rs11575934) were genotyped using the qPCR-HRM method, as described and well-developed in our laboratory (Jelassi et al., 2020).

In order to genotype the IL-12RB1 +1196 G/C (rs401502) polymorphism, a pair of primers was designed using the Primer3 tool (v.0.4.0) (http://frodo.wi.mit.edu/primer3/) (Jelassi et al., 2020). The newly designed primers, forward 5′-GCA​TTG​AAT​GGC​AGC​CTG​TG-3′ and reverse 5′-GTA​ATG​GCC​TGG​AAT​GGC​CT-3′, amplify a specific region (107bp) containing the SNP of interest. The obtained genotypes were confirmed by direct sequencing using the same primers (Jelassi et al., 2020).

For the rs11575934 polymorphism, we used the primers described by Lee et al. (2005), 5′-GGT​TAA​GTG​ACT​GGT​GCC​AAG-3′ forward and 5′-CTC​AAA​CCA​CTG​GCC​TCA​AG-3′ reverse, which amplify a 322bp sequence (Lee et al., 2005). The genotyping results were confirmed by PCR-RFLP, using PvuII enzyme (New England BioLabs, Beverly, MA, United States) at 37°C for 16 h.

### Statistical Analysis

GraphPad Prism software was used for statistical analysis (Motulsky 2016). Hardy–Weinberg equilibrium was tested among cases and controls using the chi-squared (χ2) test. Logistic regression analysis was accomplished to investigate genotype and allele frequency differences between cases and controls and calculate specific odds ratios (ORs), 95% confidential intervals (CIs), and *p* values.

## Results

### Patients’ and Healthy Subjects’ Profile

The clinical characteristics of study participants are summarized in Table 1. The sex and age distribution of subjects were very close in the patients and control groups. In fact, men represented 41.8% (46/110) and 45.4% (64/141), respectively, in the two groups, whereas mean ages were 57 ± 11.6 and 56 ± 9.6 years, respectively.

**TABLE 1:** Clinical characteristics of patients with CRC and the control group.

Variable	Case (*n* = 110)	Control (*n* = 141)
Age (mean ± SD)	57 ± 11.63	56 ± 9.55
Age < 50 years	24 (21.8%)	
Age ≥ 50 years	86 (78.2%)	
Gender		
Male	46 (41.8%)	64 (45.4%)
Female	64 (58.2%)	77 (54.6%)
Cancer site		
Colon	58 (52.7%)	-
Rectum	40 (36.4%)	-
Other	12 (10.9%)	-
TNM stage		
Stage I	9 (8.2%)	-
Stage II	38 (34.5%)	-
Stage III	52 (47.3%)	-
Stage IV	11 (10%)	-
Metastasis		
Yes	11 (10%)	-
No	99 (90%)	-
Cigarette smoking		
Yes	22 (20%)	6 (4.2%)
No	74 (67.3%)	117 (83%)
Not available	14 (12.7%)	18 (12.8%)
Alcohol consumption		
Yes	10 (9.1%)	4 (2.8%)
No	87 (79.1%)	127 (90.1%)
Not available	13 (11.8%)	10 (7.1%)

SD: standard deviation.

Different stages were observed in our group of patients, of which 8.2% of cases (9/110) were in stage I, 34.5% of cases (38/110) were in stage II, 47.3% of cases (52/110) were in stage III, and 10% of cases (11/110) were in stage IV. Metastasis was reported in 10% of cases (11/110).

The site of the tumor was the colon in 58 patients (52.7%) and the rectum in 40 patients (36.4%), and the 12 (10.9%) other patients’ locations were reported in the sigmoid.

Tobacco and alcohol consumption was low (less than 20%) in both patients and controls.

### IL-12RB1+1196G/C Polymorphism

The genotypic and allelic distributions of rs401502 SNP in case and control groups are reported in Table 2. The distribution of rs401502 SNP was in Hardy–Weinberg equilibrium (HWE) in both CRC patients and controls (*p* = 0.17 and *p* = 0.06, respectively) (Table 2). The minor allele frequencies (MAF) were 16.4% in CRC cases and 23.8% in control group. The frequencies of IL-12RB1 +1196G/C genotypes in cases and controls were as follows: GG 68.2% and 55.3%, GC 30.9% and 41.9%, and CC 0.9% and 2.8%, respectively (Table 2).

**TABLE 2:** Hardy–Weinberg test for allelic and genotypic frequencies in patients with CRC and the control group.

SNP ID		Case N=110 n (%)	HWE		Control N=141 n (%)	HWE	
			χ^2^	P		χ^2^	P
IL-12RB1 +1196G/C (rs401502)	Genotype		1.83	0.17		3.39	0.06
	GG	75 (68.2 %)			78 (55.3 %)		
	GC	34 (30.9 %)			59 (41.9 %)		
	CC	1 (0.9 %)			4 (2.8 %)		
	Allele						
	G	184 (83.6 %)			215 (76.2 %)		
	C	36 (16.4 %)			67 (23.8 %)		
IL-12RB1 +705A/G (rs11575934)	Genotype		1.26	0.26		0.042	0.83
	AA	70 (63.6 %)			69 (49 %)		
	AG	33 (30 %)			60 (42.5 %)		
	GG	7 (6.4 %)			12 (8.5 %)		
	Allele						
	A	173 (78.6 %)			198 (70.2 %)		
	G	47 (21.4 %)			84 (29.8 %)		

HWE: Hardy–Weinberg equilibrium, χ^2^: chi-squared test, P: *p*-value.

An association study of the rs401502 polymorphism with CRC risk showed a significant association in the dominant model (Table 3). In fact, we found that the frequency of the G/G genotype was 55.3% (78/141), and the frequency of G/C + C/C genotypes was 44.7% (63/141) in healthy controls, whereas these rates were 68.2% (75/110) and 31.8% (35/110), respectively, in patients.

**TABLE 3:** Allelic and genotypic distributions of the IL12RB1 +1196G/C (rs401502) polymorphism in patients with CRC and controls.

IL-12RB1 +1196G/C (rs401502)	Genotype	Case N = 110	Control N = 141	*p*-value (chi-squared test)	Or 95% CI
Co-dominant model	GG	75 (68.2%)	78 (55.3%)	0.089	
	GC	34 (30.9%)	59 (41.9%)		
	CC	1 (0.9%)	4 (2.8%)		
Dominant model (C > G)	GC + CC	35 (31.8%)	63 (44.7%)	0.038	0.5778 [0.3432 to 0.9727]
	GG	75 (68.2%)	78 (55.3%)		
Recessive model (G > C)	GG + GC	109 (99.1%)	137 (97.2%)	0.278	3.182 [0.3504 to 28.90]<
	CC	1 (0.9%)	4 (2.8%)		

N: number of samples; OR: odds ratio; CI: confidence interval.

This corresponds to a significant decrease in the risk of CRC with G/C + C/C, with OR = 0.577, 95% CI = [0.343 to 0.972], and *p* = 0.038. The frequencies of heterozygous and recessive homozygous genotypes (G/C + CC), according to the dominant model, were higher in the control group (44.7%) than those in CRC patients (31.8%), showing a potential protective effect of the rs401502 polymorphism among the Tunisian population.

On the other hand, the genetic co-dominant and recessive models showed no association with CRC risk (*p* = 0.08 and 0.27, respectively) (Table 3).

### IL-12RB1+705A/G Polymorphism

The frequency distribution of the rs11575934 SNP was in Hardy–Weinberg equilibrium (HWE) in both cases and controls (*p* = 0.26 and *p* = 0.83, respectively) (Table 2). The minor allele frequencies were 21.4% and 29.8% in CRC patients and the control group, respectively. The IL-12RB1 +705A/G polymorphism frequencies in cases and control groups were as follows: AA 63.6% and 49%, AG 30% and 42.5%, and GG 6.4% and 8.5%.

A significant association of the IL-12RB1 +705A/G polymorphism was observed with CRC cases in the genetic dominant model, as shown in Table 4. The frequency of the A/A genotype was 49.9% (69/141), and the rate of A/G + G/G genotypes was 51.1% (72/141) in healthy controls. In CRC patients, these frequencies were 64.6% (70/110) and 36.4% (40/110). This corresponds to a decreased risk of CRC in association with G/C + C/C with an OR = 0.547, 95% CI = [0.328 to 0.911], and *p* = 0.02.

**TABLE 4:** Allelic and genotypic distributions of the IL-12RB1+705 A/G polymorphism in patients with CRC and controls.

IL-12RB1 +705A/G (rs11575934)	Genotype	Case N = 110	Control N = 141	*p*-value (chi-squared test)	Or 95% CI
Co-dominant model	AA	70 (63.6%)	69 (49%)	0.066	-
	AG	33 (30%)	60 (42.5%)		
	GG	7 (6.4%)	12 (8.5%)		
Dominant model (G > A)	AG + GG	40 (36.4%)	72 (51.1%)	0.02	0.547 [0.328–0.911]
	AA	70 (64.6%)	69 (49.9%)		
Recessive model (A > G)	AA + AG	103 (93.6%)	129 (91.5%)	0.523	0.730 [0.277–1.923]
	GG	7 (6.4%)	12 (8.5%)		

N: number of samples; OR: odds ratio; CI: confidence interval.

According to the obtained results, the frequencies of the heterozygous and recessive homozygous genotypes, under the genetic dominant model, were higher in control groups (51.1%) than those in cases (36.4%). This could suggest a possible protective effect of the rs11575934 polymorphism. However, the co-dominant and recessive models did not show any significant difference (*p* = 0.66 and 0.52, respectively).

## Discussion

Colorectal cancer (CRC) is a major public health problem worldwide and in Tunisia. Many SNPs, among various genes, either involved in the immune response or the signaling pathway, have been identified as high- or low-risk variations associated with CRC. Cytokines expressed in CRC cells or in the tumor microenvironment seem to play an important role in local immunoregulation (Terzic et al., 2010; Klampfer 2011).

To the best of our knowledge, there are limited studies in a Tunisian population on whether IL-12RB1 gene polymorphisms can affect CRC risk and long-term survival in Tunisian patients with CRC.

Frequencies of rs401502 and rs11575934 SNPs were established for the first time in the general Tunisian population, using the qPCR-HRM technique (Jelassi et al., 2020). In our study, these two SNPs were genotyped by qPCR-HRM in 110 Tunisian patients with sporadic CRC. The case-control study showed a statistically significant difference between these two groups (patients and controls) for the two polymorphisms rs401502 and rs11575934 with OR = 0.577, 95% CI = [0.343 to 0.972], *p* = 0.038 and OR = 0.547, 95% CI = [0.328 to 0.911], *p* = 0.02, respectively. Frequencies of the mutant allele the heterozygous and recessive homozygous genotypes for two target SNPs were higher in the control group than those in patients with CRC. This suggests that these mutations did not present a risk factor for sporadic CRC and that they would rather be associated with a potential protective effect. We observed that genetic studies increasingly show that mutations have a potential protective role in certain multifactorial diseases (Yamamoto-Furusho et al., 2011), in particular cancerous pathologies (Tong et al., 2012; Núñez-Marrero 2020). Globally, association studies of these SNPs with sporadic CRC are rare. Our results agree with those already reported by Chubb et al. that have shown the importance of IL-12RB1 as a candidate gene for inherited CRC (Chubb et al., 2016a). Recently, a GWAS study identified three SNPs, c.94C > T (p.Gln32Ter), c.1624C > T (p.Gln542Ter), and c.1237T > C (p.Cys413Arg), linked to inherited CRC in the frame of a family study (Belhadj et al., 2020). These results were based on previous studies that identified IL-12RB1 as a potential candidate gene for hereditary CRC due to its biological importance (Chubb et al., 2016a).

The IL-12RB1 polymorphisms have been explored in other cancers including cervical cancer (Hussain et al., 2013); breast cancer (Quan et al., 2014); esophageal cancer (Tao et al., 2012); and gastric cancer (Vogelaar et al., 2015). The role of mutations in the IL-12RB1 gene in the carcinogenesis process and its consequences is controversial. Núñez-Marrero et al. suggested that certain polymorphisms (such as rs3761041, rs401502, and rs404733 SNPs) increase the risk of breast cancer through a loss-of-function effect, thereby leading to a reduced transduction signal of the IL-12 cytokine. Indeed, certain mutations in the IL-12RB1 gene generate an inactive IL-12Rβ1 protein. This deficit abolishes both IL-12 and IL-23 signaling pathways (Núñez-Marrero 2020). On the other hand, still concerning breast cancer, certain variations (in particular the rs438421 SNP) would be protective by improving the activity of the IL-12R receptor, thus promoting the production of IFN-γ especially in cytotoxic T cells (van de Vosse et al., 2013; Núñez-Marrero 2020). Furthermore, beyond the mutations favorable to the development of the disease or those which would be protective of it, rare variations and other common ones with an unknown functional effect have been reported for IL-12RB1 (van de Vosse et al., 2013). Many studies have investigated the involvement of certain SNPs within genes encoding cytokines and their receptors such as IL-6, IL1β, IL-1RN, IL-10, IL17, and TNFα. Indeed, interleukin-6 (IL-6) and tumor necrosis factor (TNF) are the most widely studied cytokines in CRC and other malignancies. These two cytokines actively participate in the signal transduction of the STAT3 pathway and NF-κB, respectively. These cytokines also promote tumor progression by enhancing proliferation, invasion, and resistance to apoptosis (Uchiyama et al., 2012; West et al., 2015).

Concerning the IL-12RB1 gene, the target SNPs lead to the absence of the IL-12Rβ1 expression on the surface of activated T and NK cells or, more rarely, they give rise to a non-functional IL12RB1 surface protein. This alteration of the protein expression could alter the inflammatory processes, leading to a protective effect through a decrease in chronic inflammation. In all cases, additional proteomics and functional studies are necessary in order to clarify their precise impact.

## Conclusion

This study shows for the first time an association between two SNPs in IL-12RB1 gene (rs401502 and rs11575934) and the sporadic CRC, with a potential protective effect. Furthermore, it would be interesting to study the correlation between these polymorphisms and protein expression.

## Data Availability

The datasets presented in this study can be found in online repositories. The names of the repository/repositories and accession number(s) can be found in the article/supplementary material.
